# Associations between dental problems and underweight status among rural women in Burkina Faso: results from the first WHO Stepwise Approach to Surveillance (STEPS) survey

**DOI:** 10.1017/S1368980021004080

**Published:** 2022-08

**Authors:** Jeoffray Diendéré, Augustin Nawidimbasba Zeba, Sibraogo Kiemtoré, Olivier Ouahamin Sombié, Philippe Fayemendy, Pierre Jésus, Athanase Millogo, Aly Savadogo, Halidou Tinto, Jean-Claude Desport

**Affiliations:** 1 Research Institute for Health Sciences, 399, Avenue de la Liberté, 01 BP 545, Bobo-Dioulasso, Burkina Faso; 2 Gynecologic and Obstetrics’ Department, Yalgado Ouédraogo University Hospital, Ouagadougou, Burkina Faso; 3 Nutrition Unit, University Hospital of Limoges, Limoges, France; 4 INSERM, U1094, Tropical Neuroepidemiology, Limoges, France; 5 University of Limoges, UMR_S 1094, Tropical Neuroepidemiology, Institute of Neuroepidemiology and Tropical Neurology, CNRS FR 3503 GEIST, Limoges, France; 6 Medicine Department, Souro Sanou University Hospital, Bobo-Dioulasso, Burkina Faso; 7 Département de Biotechnologie Alimentaire, Centre de Recherche en Sciences Biologiques, Alimentaires et Nutritionnelles (CRSBAN), Joseph Ki-Zerbo University, Ouagadougou, Burkina Faso; 8 Research Institute for Health Sciences, Clinical Research Unit of Nanoro, Nanoro, Burkina Faso

**Keywords:** Rural women, Underweight, Dental problems, Prevalence, Risk factors, Burkina Faso

## Abstract

**Objective:**

To explore the relationships between dental problems and underweight status among rural women in Burkina Faso by using nationally representative data.

**Design:**

This was a cross-sectional secondary study of primary data obtained by the 2013 WHO Stepwise Approach to Surveillance survey conducted in Burkina Faso. Descriptive and analytical analyses were performed using Student’s *t* test, ANOVA, the *χ*^2^ test, Fisher’s exact test and logistic regression.

**Setting:**

All thirteen Burkinabè regions were categorised using quartiles of urbanisation rates.

**Participants:**

The participants were 1730 rural women aged 25–64 years.

**Results:**

The prevalence of underweight was 16·0 %, and 24·1 % of participants experienced dental problems during the 12-month period. The women with dental problems were more frequently underweight (19·9 % and 14·7 %; *P* < 0·05) and had a lower mean BMI (21·1 ± 3·2 and 21·6 ± 3·7 kg/m^2^, *P* < 0·01) than those without dental problems. More risk factors for underweight were observed in less urbanised regions among elderly individuals (> 49 years old) and smokeless tobacco users. Age > 49 years, professions with inconsistent income, a lack of education, smokeless tobacco use and low BMI were factors that were significantly associated with dental problems, while residency in a low-urbanisation area was a protective factor.

**Conclusion:**

The prevalence of underweight in rural Burkinabè women is among the highest in sub-Saharan Africa, and women with dental problems are more frequently affected than those without dental problems. Public health measures for the prevention of these disorders should specifically target women aged over 49 years and smokeless tobacco users.

Undernourishment is persistent in low- and middle-income countries, particularly in sub-Saharan Africa (SSA)^(1)^. It is usually more common in rural areas than in urban areas, likely due to poverty and food scarcity in rural areas^(2–5)^. Females are more frequently affected by undernourishment than males^(6)^, particularly in rural areas^(7)^. In Burkina Faso, a country in SSA, rural residents account for 77·3 % of the population, and 52·2 % are female^(8)^. In a previous study on nutritional status and swallowing disorders in the Burkinabe hospital setting, females were seven times more likely to be undernourished than males^(9)^. This finding suggests that females are more susceptible to undernourishment, especially when health problems exist. Furthermore, dental problems may affect the preparatory phase of the swallowing process^(10)^, creating swallowing difficulties with possible impacts on nutritional status. Data from SSA about dental problems in children^(11)^ and elderly people are scarce^(12)^, and data on women focus mostly on pregnant women^(13,14)^. Among Ugandan pregnant women, 30 % experienced at least one oral-related impact on performance of daily activities in the 6 months preceding a cross-sectional survey in 2006; the frequency of impact on eating was 24·4 %, on speaking was 9·1 %, and on smiling was 5·1 %^(13)^. Among elderly Cameroonian individuals, barriers to dental health care include financial difficulties (67·8 %), lack of awareness (25·7 %) and distance to the nearest clinic (6·5 %)^(12)^. Dental problems (such as tooth loss) may induce impairments in mastication^(15)^ and swallowing and may decrease the BMI^(16)^. The relationship between undernourishment and dental problems in a population-based study, particularly in non-pregnant women living in rural Burkina Faso, has not yet been examined. The first national survey using the WHO Stepwise Approach to Surveillance (WHO STEPS) was a population-based study that collected variables related to oral health (including dental problems) and nutritional status in Burkina Faso. The aim of this paper is to explore the relationship between dental problems and underweight status among rural women in Burkina Faso by analysing nationally representative data.

## Methods

### Study design

A secondary cross-sectional analysis was performed using data from the WHO STEPS^(17,18)^ survey conducted in Burkina Faso. The current study is a recommended tool for surveillance of chronic diseases and their risk factors in WHO member countries. The survey is a standardised method to collect, analyse and disseminate data. It is a sequential process that starts with gathering key information about risk factors with a questionnaire; subsequently, simple physical measurements and blood samples for biochemical analysis are collected. The WHO STEPS includes a representative sample of the study population, which allows the results to be generalisable to the entire population^(18)^.

### Study population

The study population was adults of both sexes aged 25 to 64 years who had been living in Burkina Faso for at least 6 months on the day of the survey. We analysed the data of only women living in rural areas.

### Sample size, data collection and women included in the analyses

The total sample size calculation and the data collection process throughout the country have been described elsewhere^(19,20)^. The National Institute for Statistics and Demography (Institut National de la Statistique et de la Démographie, INSD) of Burkina Faso provided maps and data on enumeration areas and their number of households which informed the representative sampling process. The INSD used data from the latest *General Census of Population and Housing* (2006) and updated in 2010 during the Demographic and Health Survey in Burkina Faso to define the enumeration areas or clusters. More details on the enumeration areas can be found elsewhere^(8)^. The sample size calculation in the WHO STEPS non-communicable disease risk factor survey was based on the prevalence of hypertension (primary outcome). The nationally representative sample size, based on 20 % non-response, was estimated as 4785 (rounded up to 4800) adults aged 25–64 years. Since the national adult prevalence of underweight is unknown, if it is assumed to be 50 %, the sample size would be smaller than 4800.

A stratified three-stage cluster proportional to the size sampling was used to select participants. The sample was stratified to provide adequate representation of both rural and urban residence. An excel spreadsheet was used to draw households from each selected cluster. One individual aged 25–64 years was randomly selected from each household using the Kishmethod^(21)^.

The data collection team consisted of supervisors and interviewers. The supervisors were statisticians, epidemiologists and clinicians. The interviewers were nurses and medical students at the end of their training paths and who had proven experience in population surveys. The field staff was trained to collect the data using standard tools and methods. They were trained over a period of 5 d and participated in a field pre-test of the study instruments. Data were collected using a questionnaire and physical measurements. Data collection was conducted from 3 September to 24 October 2013. The data were collected using standardised WHO STEPS questionnaires input into laptop computers. Household socio-demographic information was recorded via face-to-face interviews in the language spoken by the participant after blood pressure and anthropometric measurements were collected.

After data collection, 105 individuals were not eligible or had invalid data regarding sex. Of the remaining population, 2257 were men, 518 were urban women and 1920 were rural women. Our analyses included only non-pregnant rural women with complete socio-demographic, lifestyle and nutritional data and with responses on items used to screen for dental problems. In total, 1730 rural women were included in the analyses.

### Variables of interest extracted from the Stepwise Approach to Surveillance survey database

The participants’ demographic variables included age (25–64 years), marital status (grouped into (i) married or cohabitating; (ii) single; (iii) education level (grouped into i) no formal schooling; (iv) primary school or higher; (v) occupation (grouped into i) public or private formal employment or self-employed; (vi) employment with inconstant or (vii) irregular income, such as students, housekeepers or unemployed. We also reported women living in households with or without at least one member aged ≥ 18 years. Anthropometric characteristics were weight (kg), height (m), BMI (weigh/height^2^, kg/m^2^) and waist circumference (cm). Height was measured to the nearest 0·1 cm using a stadiometer (SECA 214) on a subject without shoes, while weight was measured to the nearest 0·1 kg with a personal scale (SECA 813) on a lightly clothed subject without shoes. Waist circumference was measured to the nearest 0·1 cm (as per WHO recommendations) with a measuring tape (SECA 203) at the midpoint between the last rib and the iliac crest, with the subjects standing upright and breathing normally. BMI < 18·5 kg/m^2^ was defined as underweight^(22)^. A mobile device (CardioChek™ 1708 PA) was used for the biochemical measurements. Blood pressure (in mmHg, systolic and diastolic blood pressure values) was measured three times, with their mean value being used in the analysis. All measurement devices were provided by the WHO. Physical measurements were carried out on the same day. Lifestyle factors assessed during the interviews were self-reported smokeless (chewing, snorting) tobacco use over the past year and current alcohol consumption over the past month recorded. Based on the quantity of alcohol drunk during the past month, alcohol drinkers were classified as mild/moderate drinkers if they currently consumed six standard drinks or less and binge drinkers if they consumed more than six standard drinks. A standard drink was defined as the amount of alcohol in one glass of beer, one glass of wine or one shot of spirits. In addition, pictures illustrating local containers and volumes of standard drink of beer, wine and spirits glasses were showed to the respondents. Dental problems were also recorded by a self-reporting method and defined as the occurrence of any of the following in the past 12 months: (i) difficulty chewing food; (ii) difficulty pronouncing words or (iii) tooth/mouth pain or discomfort.

### Categorisation of the country’s urbanisation gradient

**Fig. 1:**
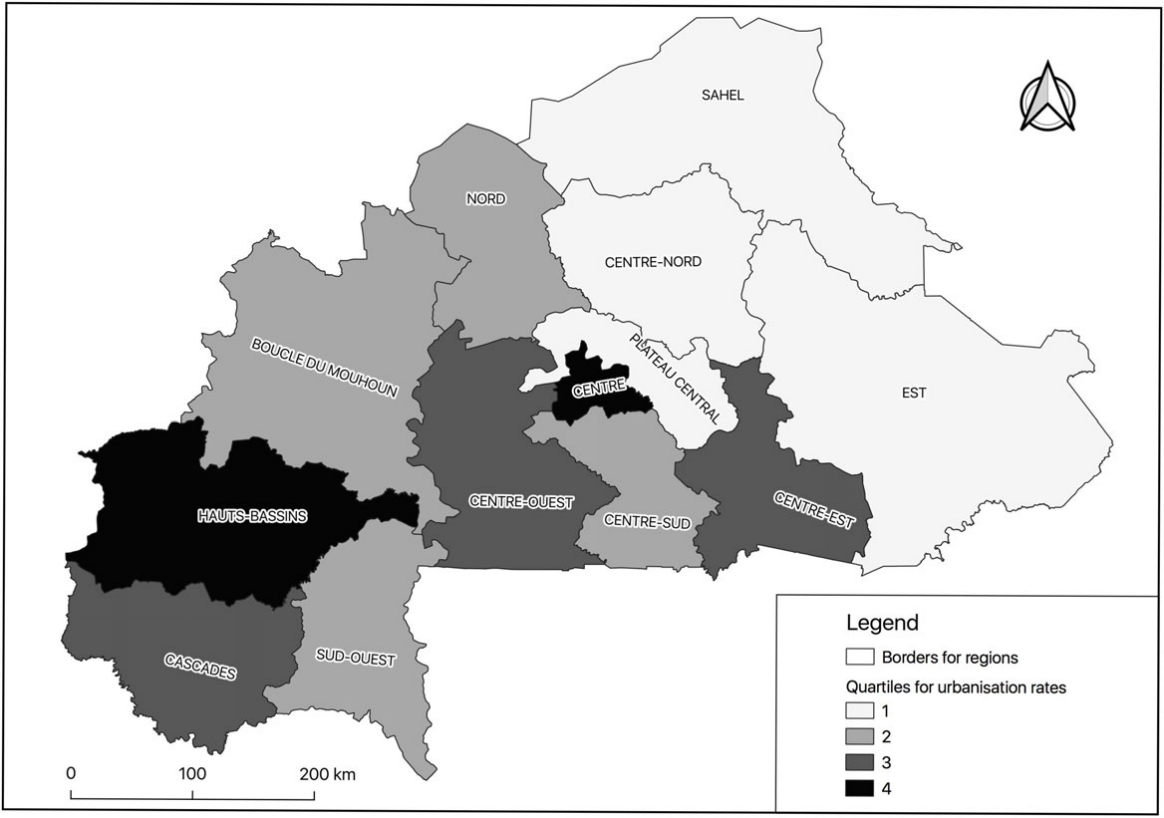
Urbanization gradient for the thirteen Burkinabe administrative regions (respective values for quartiles of the urbanisation rate are 8·1 %, 11·8 % and 19·3 %). This categorisation resulted in rural locations attached to regions with the lowest level of urbanisation in the first quartile and those with the highest level in the fourth quartile

Burkina Faso is divided into thirteen administrative regions, each with a specific rate of urbanisation. Since urbanisation process influences the nutritional status of subjects, we categorised the regions of the country into four subgroups according to their level of urbanisation. The regions are classified by quartiles according to the regional urbanisation rate. The national mean rate is 23·3 % (minimum = 6·6 %, maximum = 85·4 %)^(8)^, and the quartile values are 8·1, 11·8 and 19·3 %. Four regions are included in the first quartile (Q1) and second quartile (Q2), three regions in the third quartile (Q3) and two regions (‘centre’ and ‘Hauts-Bassins’) in the fourth quartile (Q4) (Fig. 1). The political capital Ouagadougou (in the ‘centre’ region, with 46·4 % of the country’s urban dwellers) and the economic capital Bobo-Dioulasso (within the ‘Hauts-Bassins’ region, with 15·4 % of the country’s urban dwellers) are in the last quartile^(8)^. These two regions are densely urbanised. This categorisation suggests that the rural locations attached to the regions with low levels of urbanisation and thus ranked in the first quartiles reflect those geographical spaces less influenced by the urbanisation process.

### Statistical analyses

StataCorp.™ Stata Statistical Software for Windows (Version 14.0) was used to analyse the data. The quantitative variables are expressed as the means ± SD, and the qualitative variables are expressed as percentages (%) with 95 % CI. Student’s *t* test or ANOVA was used to compare quantitative variables, and the *χ*^2^ test and Fisher’s exact test were used to compare categorical variables. Logistic regression analysis was performed to identify clinical and lifestyle factors associated with underweight status after adjustment for socio-demographic features. The second analysis considered dental problems as a dependent factor. All independent variables with a *P*-value <0·20 in the univariate analyses were included in the final model. The final model was established by backward elimination, i.e. the progressive elimination of non-significant factors by decreasing the order of significance. After grouping the 1730 observations into ‘deciles of risk’ in which observations were partitioned into ten groups, the Hosmer-Lemeshow test was performed to determine the goodness-of-fit of the logistic regression models. A *P*-value >0·05 in the Hosmer-Lemeshow *χ*^2^ test was considered significant. Excluding the Hosmer-Lemeshow test, for all analyses, a *P*-value <0·05 % was considered significant.

### Ethical considerations

The protocol of the WHO STEPS survey was approved by the Ethics Committee for Health Research of the Ministry of Health of Burkina Faso (deliberation no: 2012-12092; 5 December 2012). Written informed consent was systematically obtained from each participant in the STEPS survey.

## Results

The mean age in the sample was 37·8 ± 10·9 years, and other socio-demographic characteristics are presented in Table 1. The prevalence of underweight was 16·0 % (95 % CI 14·3, 17·8), and 24·1 % (95 % CI 22·1, 26·2) of women experienced dental problems during the past 12 months. Women with dental problems were more frequently underweight than those without dental problems (19·9 % and 14·7 %, *P* < 0·05). Regarding lifestyle factors, 13·8 % (95 % CI 12·2, 15·5) were smokeless tobacco users, 14·4 % (95 % CI 12·8, 16·1) were moderate alcohol users and 10·2 % (95 % CI 8·8, 11·7) were binge drinkers (results not shown).

**Table 1:** Socio-demographic characteristics of rural women who were/were not underweight and those with/without dental problems

	Overall			Underweight			Not underweight			*P*	With dental problems			Without dental problems			*P*
	*n* 1730			*n* 276			*n* 1454				*n* 417			*n* 1313			
	*n*	%	95 % CI	*n*	%	95 % CI	*n*	%	95 % CI		*n*	%	95 % CI	*n*	%	95 % CI	
Urbanisation rate										0·001							0·001
Q1	412	23·8	21·8, 25·9	77	27·9	22·7, 33·6	335	23·0	20·9, 25·3		84	20·1	16·4, 24·3	328	25·0	22·6, 27·4	
Q2	532	30·8	28·6, 33·0	77	27·9	22·7, 33·6	455	31·3	28·9, 33·7		147	35·3	30·7, 40·0	385	29·3	26·9, 31·9	
Q3	581	33·6	31·4, 35·9	106	38·4	32·6, 44·4	475	32·7	30·3, 35·1		119	28·5	24·2, 33·1	462	35·2	32·6, 37·8	
Q4	205	11·8	10·4, 13·5	16	5·8	3·3, 9·2	189	13·0	11·3, 14·8		67	16·1	12·7, 19·9	138	10·5	8·9, 12·3	
Age range (years)										0·001							0·001
25–34	809	46·8	44·4, 49·1	101	36·6	30·9, 42·6	708	48·7	46·1, 51·3		146	35·0	30·4, 39·8	663	50·5	47·7, 53·2	
35–49	592	34·2	32·0, 36·5	94	34·1	28·5, 40·0	498	34·2	31·8, 36·8		163	39·1	34·4, 44·0	429	32·7	30·1, 35·3	
> 49	329	19·0	17·2, 20·9	81	29·3	24·0, 35·1	248	17·1	15·2, 19·1		108	28·9	21·8, 30·4	221	16·8	14·8, 19·0	
Marital status										0·02							0·06
Married/cohabitating	1553	89·8	88·2, 91·2	237	85·9	81·2, 89·8	1316	90·5	88·9, 92·0		364	87·3	83·7, 90·3	1189	90·6	88·8, 92·1	
Single	177	10·2	8·8, 11·8	39	14·1	10·2, 18·8	138	9·5	8·0, 11·1		53	12·7	9·7, 16·3	124	9·4	7·9, 11·1	
Education level										0·01							0·001
No formal education	1558	90·1	88·6, 91·4	260	94·2	90·8, 96·7	1298	89·3	87·6, 90·8		393	94·2	91·6, 96·3	1165	88·7	86·9, 90·4	
Primary school or more	172	9·9	8·6, 11·4	16	5·8	3·3, 9·2	156	10·7	9·2, 12·4		24	5·8	3·7, 8·4	148	11·3	9·6, 13·1	
Occupation										0·12							0·06
Employed/self-employed	1002	57·9	55·6, 60·3	148	53·6	47·5, 59·6	854	58·7	56·2, 61·3		225	54·0	49·0, 58·8	777	59·2	56·5, 61·9	
Others (students + household-keepers + unemployed)	728	42·1	39·7, 44·4	128	46·4	40·4, 52·5	600	41·3	38·7, 43·8		192	46·0	41·2, 51·0	536	40·8	38·1, 43·5	
Having at least one family member aged ≥ 18 years										0·048							0·52
Yes	1286	74·3	72·2, 76·4	84	30·4	25·1, 36·2	360	24·8	22·6, 27·1		315	75·5	71·1, 79·6	971	74·0	71·5, 76·3	
No	444	25·7	23·6, 27·8	192	69·6	63·8, 74·9	1094	75·2	72·9, 77·4		102	24·5	20·4, 28·9	342	26·0	23·7, 28·5	

Q1, Q2, Q3, Q4: first, second, third and fourth quartiles.

Nutritional and clinical features in women with and without dental problems are compared in Table 2. Women with dental problems had lower weight (55·0 ± 8·9 kg and 56·9 ± 10·5 kg, *P* < 0·001) and BMI (21·1 ± 3·2 and 21·6 ± 3·67 kg/m^2^; *P* < 0·01).

**Table 2:** Nutritional and clinical features according to the rate of urbanisation, the presence or absence of dental problems and nutritional status

			Urbanisation rate gradient									Without or with dental problems					Nutritional state				
	Overall		Q1		Q2		Q3		Q4		*P*	Without dental problems		With dental problems		*P*	Not underweight		Underweight		*P*
	*n* 1730		*n* 412		*n* 532		*n* 581		*n* 205			*n* 1313		*n* 417			*n* 1454		*n* 276		
	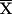	sd	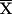	sd	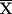	sd	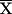	sd	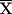	sd		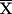	sd	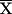	sd		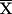	sd	µ	sd	
Weight (kg)	56·4	10·2	55·9	11·1	56·4	9·9	55·4	8·9	60·4	11·7	0·0001	56·9	10·5	55·0	8·9	0·001	58·3	9·8	46·4	4·9	0·0001
BMI (kg/m^2^)	21·5	3·6	21·2	3·8	21·6	3·3	21·1	3·4	23·0	3·9	0·0001	21·6	3·67	21·1	3·2	0·004	22·3	3·3	17·4	1·1	0·0001
Waist circumference (cm)	76·2	11·6	75·6	14·3	75·3	9·6	75·9	9·5	80·5	14·5	0·0001	76·2	11·9	76·2	10·8	0·95	77·3	11·4	70·3	10·9	0·0001
Systolic blood pressure (mmHg)	120·0	16·7	120·7	16·8	119·2	17·2	119·6	16·0	122·4	16·9	0·095	119·7	15·7	121·4	19·5	0·065	120·2	16·7	119·4	16·7	0·46
Diastolic blood pressure (mmHg)	76·8	10·0	75·9	10·0	75·3	9·7	77·5	9·7	80·7	10·7	0·0001	76·8	10·0	76·9	10·2	0·76	76·9	10·1	76·4	9·8	0·48
†Nutritional state											0·0001*					0·025*					…
Underweight																					
%	16·0		18·7		14·5		18·2		7·8			14·7		19·9		0·012	…		…		
95 % CI	14·3, 17·8		15·0, 22·8		11·6, 17·8		15·2, 21·6		4·5, 12·4			12·8, 16·7		16·2, 24·1			…		…		
Normal																					
%	70·9		70·9		71·8		72·3		64·9			71·2		70·0		0·64	…		…		
95 % CI	68·7, 73·1		66·2, 75·2		67·8, 75·6		6·8, 7·6		57·9, 71·4			68·7, 73·6		65·4, 74·4			…		…		
Overweight																					
%	10·1		7·0		11·3		7·4		21·0			10·8		7·9		0·087	…		…		
95 % CI	8·7, 11·6		4·8, 10·0		8·7, 14·3		5·4, 9·8		15·6, 27·2			9·2, 12·6		5·5, 10·9			…		…		
Obese																					
%	3·0		3·4		2·4		2·1		6·3			3·3		2·2		0·25	…		…		
95 % CI	2·3, 3·9		1·9, 5·6		1·3, 4·1		1·1, 3·6		3·4, 10·6			2·4, 4·3		1·0, 4·1			…		…		

Q1, Q2, Q3, Q4: first, second, third and fourth quartiles; 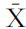 : mean.

* Global *P*-value.

† Nutritional status was expressed as % (confidence interval (CI 95 %) at 95 %).

Living in a region with a lower rate of urbanisation increased the risk of underweight if one looks at results related to Q3 and Q1, but the results are NS for Q2 in multivariate analysis (Table 3). This risk also affected women > 49 years old (aOR = 1·82; (95 % CI 1·29, 2·57)) and smokeless tobacco users (aOR = 2·17; (95 % CI 1·54, 3·06)).

**Table 3:** Factors associated with underweight among women in rural Burkina Faso (*n* 1730)

Factors	Univariate analysis			Multivariate analysis		
	cOR	95 % CI	*P*	aOR	95 % CI	*P*
Urbanization rate (%, in quartiles)						
Q4	1			1		
Q3	2·64	1·52, 4·56	0·001	2·44	1·40, 4·25	0·002
Q2	2·00	1·14, 3·52	0·016	1·66	0·93, 2·95	0·085
Q1	2·72	1·54, 4·79	0·001	2·45	1·38, 4·35	0·002
Age range (years)						
25–34	1			1		
35–49	1·32	0·98, 1·79	0·07	1·23	0·90, 1·68	0·19
> 49	2·29	1·65, 3·17	0·0001	1·82	1·29, 2·57	0·0001
Marital status: single *v*. married/cohabitating (ref)	1·57	1·07, 2·30	0·021	1·15	0·75, 1·78	0·47
Having at least one family member aged ≥ 18 years: yes *v*. no (ref)	1·33	1·00, 1·76	0·048	1·24	0·93, 1·67	0·60
Education level: no education *v*. primary or more (ref)	1·95	1·15, 3·32	0·014	1·52	0·88, 2·61	0·11
Occupation: others* (ref) *v*. employed/self-employed	1·23	0·95, 1·59	0·12	1·21	0·93, 1·58	0·44
Current alcohol use						
Non-user	1			1		
Moderate user	1·56	1·11, 2·19	0·01	1·30	0·91, 1·86	0·15
Binge drinker	1·18	0·78, 1·80	0·43	1·09	0·70, 1·70	0·71
Smokeless tobacco consumption: yes *v*. no (ref)	2·47	1·80, 3·39	0·0001	2·17	1·54, 3·06	0·0001
Dental problems: yes *v*. no (ref)	1·44	1·08, 1·92	0·01	1·29	0·96, 1·74	0·094
Systolic blood pressure (mmHg)	> 0·99	0·99, 1·01	0·46			
Diastolic blood pressure (mmHg)	> 0·99	0·98, 1·01	0·48			

cOR: crude OR; aOR: adjusted OR.

Others*: includes professions with inconstant income, i.e. students, housekeepers, unemployed.

The goodness-of-fit test of this logistic regression reports the *χ*^2^ of Hosmer-Lemeshow at seven degrees of liberty of 5 08, with a *P*-value of 0 65.

Table 4 shows the associations with dental problems in the logistic regression analysis. Belonging to a region with a lower rate of urbanisation reduced the risk for dental problems by 36 % to 60 % (across quartiles of the urbanisation rate), whereas this risk was increased in women aged 35–49 years (aOR = 1·57; (95 % CI 1·20, 2·04)), aged > 49 years old (aOR = 1·76; (95 % CI 1·29, 2·41)), without an education (aOR = 1·65; (95 % CI 1·04, 2·62)), and working in professions with inconstant income (aOR = 1·34; (95 % CI 1·05, 1·70)) and in smokeless tobacco users (aOR = 2·38; (95 % CI 1·74, 3·24)). A decrease of one unit in BMI (in kg/m^2^) was linked to an increase in the risk of dental problems of 4 %. Regarding the goodness-of-fit test for each logistic regression model, the Hosmer-Lemeshow *χ*^2^ test had a *P*-value over 0·05.

**Table 4:** Factors associated with dental problems among women in rural Burkina Faso (*n* 1730)

Factors	Univariate analysis			Multivariate analysis		
	cOR	95 % CI	*P*	aOR	95 % CI	*P*
Urbanization rate (%, in quartiles)						
Q4	1			1		
Q3	0·53	0·37, 0·76	0·0001	0·44	0·30, 0·64	0·0001
Q2	0·77	0·56, 1·11	0·18	0·64	0·44, 0·92	0·017
Q1	0·53	0·36, 0·77	0·001	0·40	0·27, 0·60	0·0001
Age range (years)						
25–34	1			1		
35–49	1·73	1·34, 2·22	0·0001	1·57	1·20, 2·04	0·001
> 49	2·22	1·66, 2·97	0·0001	1·76	1·29, 2·41	0·0001
Occupation: others* (ref) *v*. employed/self-employed	1·24	0·99, 1·54	0·06	1·34	1·05, 1·70	0·017
Marital status: single *v*. married/cohabiting (ref)	1·40	0·99, 1·97	0·06	1·10	0·75, 1·61	0·62
Having at least one family member aged ≥ 18 years: yes *v*. no (ref)	0·92	0·71, 1·19	0·52			
Education level: no education or primary school *v*. secondary or more (ref)	2·08	1·33, 3·25	0·001	1·65	1·04, 2·62	0·032
Current alcohol use						
Non-user	1			1		
Moderate user	1·27	0·94, 1·73	0·12	0·92	0·67, 1·28	0·64
Binge drinker	1·18	0·82, 1·68	0·18	0·87	0·59, 1·28	0·48
Smokeless tobacco consumption: yes *v*. no (ref)	2·99	2·25, 3·97	0·0001	2·38	1·74, 3·24	0·0001
Waist circumference (cm)	> 0·99	0·99, 1·01	0·95			
BMI (kg/m^2^)	0·95	0·92, 0·98	0·004	0·96	0·93, 0·99	0·017
Systolic blood pressure	1·01	0·99, 1·01	0·07	>1·00	0·99, 1·01	0·62
Diastolic blood pressure	>1·00	0·99, 1·01	0·76			

cOR: crude OR; aOR: adjusted OR.

* Others: included professions with inconstant income, i.e. students, housekeepers and unemployed.

The goodness-of-fit test of this logistic regression reports the *χ*^2^ of Hosmer-Lemeshow at eight degrees of liberty of 10 32, with a *P*-value of 0 25.

## Discussion

### Prevalence of underweight and associated factors

The prevalence of underweight in rural Burkinabe women (16·0 %) was close to the prevalence reported in rural women in Ghana (13·1 %)^(23)^ and Uganda (16 % in northern socio-politically troubled areas)^(24)^. In contrast, a low prevalence of 11·2 % was reported in rural women in Kenya^(25)^, 10·9 % in Angola^(26)^, 7·8 % in Zambia^(27)^, 7·0 % in Tanzania^(28)^ and 2·6 % in Nigeria^(29)^. The level of underweight status is low when a country’s food security is high^(30)^. Women with dental problems had a significantly higher percentage of underweight in the bivariate analysis than those without dental problems. However, no significant association was observed between dental problems and underweight when the regression analysis was conducted. Poor oral health may lead to impaired masticatory function with swallowing impairment and possible food intake avoidance^(31,32)^. Low nutritional status was found to be associated with poor oral health^(33,34)^, and the association between underweight status and tooth loss was demonstrated among Korean adults^(35)^. Undernourishment decreased significantly from the lower to the higher urbanisation regions (18·7 % to 7·8 %; *P* < 0·05) (Table 2, Fig. 1). In this Sahelian region, a change in the underweight rate might mirror food scarcity attributable to geographic factors, including rainfall deficiency^(36)^. The influence of rainfall on female nutritional status was established in Uganda^(24)^. In addition, the number of births was higher in the less urbanised regions in Burkina Faso (the total fertility rate was 7·8 in the ‘east region’ included in Q1 and 4·1 in the ‘centre region’ included in Q4)^(8)^, and an association between parity ≥ 5 and household food insecurity was found in Ethiopia (aOR = 10·76, (95 % CI 1·38, 84·28))^(37)^. The rate of underweight is higher in rural areas than in urban areas, probably because of the lower purchasing power in rural areas, resulting in less food availability^(38)^. The mean age of the study participants was 37·8 ± 10·9 years. There was no significant relationship between underweight and dental problems in the regression analysis. Our finding is similar to one involving post-stroke Burkinabè patients with mean age 60·5 ± 14·2 years^(9)^. However, significant relationships have frequently been observed among older people^(39)^, as in Malaysia (mean age, 73·4 ± 7·3 years)^(40)^or Brazil (mean age, 72·7 ± 5·8 years)^(41)^.

Women aged > 49 years have a high risk of underweight (aOR = 1·82; *P* < 0·001) (Table 3), as reported by Schramm *et al.* in Ugandan women of perimenopausal age (15–19 years, significant aOR = 3·25 ((45–54 years), 3·67 (54–64 years) and 6·97 (≥ 65 years))^(24)^. In SSA, females are considered by humanitarian organisations or non-governmental organisations^(42)^ to be a group vulnerable to food insecurity and are usually included as a target group for food aid and nutrition interventions, particularly in maternal and child health programmes^(43)^. However, older adult women, especially those who are menopausal, no longer seem to be a primary target for these aid programmes and nutrition interventions and seem to be excluded from food aid programmes, resulting in increasing undernourishment. Among menopausal women who were followed for 2 years, 57·5 % lost at least one tooth, with a mean tooth loss per person of 1·8 ± 2·8^(44)^. The odds of the loss of four or more teeth increased in the age groups of 35–44 and 45–64 years (compared with those aged 20–34 years) in women in São Paulo^(45)^. A reduction in the number of functional dental units can result in impairments in chewing or mastication^(46)^, resulting in eating difficulties. Tooth loss can lead to reduced nutrient intake and low serum albumin levels^(47,48)^. Dental caries can also lead to masticatory dysfunction with reduced food intake^(49,50)^.

Smokeless tobacco users were at high risk for undernourishment (aOR = 2·17; (95 % CI 1·54, 3·06)), as previously found in rural Burkinabe women, among whom tobacco chewing was associated with decreased BMI^(51)^, and in rural south India^(52)^. Smokeless tobacco contains nicotine, which is a major appetite suppressant^(53)^ and mediates inadequate food intake, leading to undernourishment. Tobacco is also known to increase resting energy expenditure by central mediation^(54)^ and consequently increases total energy expenditure.

### Factors associated with dental problems

Nearly one-quarter of rural women experienced dental problems 12 months prior to the data collection, similar to the results of Pau *et al.*, in which 12–40 % of adult community dwellers in the UK were affected by dental pain^(55)^. The odds of experiencing dental problems in less urbanised areas were approximately 40 % less (Table 4) than in urbanised areas. Psychological stress is favourable for dental health impairment^(56)^, and living in a region with a low urbanisation rate may reduce stress levels^(57)^. Furthermore, food preparation techniques^(58)^ or food components^(59)^ can affect the cariogenicity of a food. Gondivkar *et al.*^(32)^ reported that dental problems, especially pain, were associated with unhealthy intake patterns (aOR: 1·27–1·81), including the consumption of soda, fruit juice, diet soda, frozen desserts, sweet rolls, candy, white rice/pasta, starchy vegetables, French fries/chips and cereal^(60)^. Unfortunately, the data collected in Burkina Faso did not include specific dietary profiles for each region and therefore did not allow us to assess these relationships.

The mean BMI was lowest in rural women with dental problems (21·1 and 21·6 kg/m^2^; *P* < 0·01) (Table 2). In the multivariable analysis, we found that the higher the BMI was, the lower the occurrence of dental problems (aOR = 0·96; *P* < 0·05) (Table 4). Studies highlighting the impact of oral health on nutritional status have usually focused on elderly individuals^(61)^. Dental problems were found to be associated with oropharyngeal dysphagia (which may result in reduced food intake)^(62)^, and tooth loss and infrequent food intake were associated with weight loss^(63)^.

Dental problems increased with age, and women aged > 49 years had the highest risk (aOR = 1·76; *P* < 0·001) (Table 4). The authors speculate that after menopause, women are more susceptible to periodontal disease because of oestrogen deficiency, resulting in bone loss and inflammatory processes^(64)^. Meurman *et al.* reported that peri- and postmenopausal problems included dry mouth and burning pain in the mouth (glossodynia), which in turn might increase the occurrence of oral mucosal and dental diseases^(65)^.

Women working in professions with inconstant income (students, housekeepers and unemployed) had an increased risk of dental problems (Table 4). These problems might also be related to psychological distress^(66)^ mediated by joblessness or poverty.

A lack of education was a risk factor for dental problems (Table 4), in accordance with the study by Umer *et al.* in West Virginia (USA), which showed that women with a high school education were more likely to undergo dental cleanings^(67)^. This behaviour may be favourable for dental health.

Smokeless tobacco use was a risk factor for dental problems, in accordance with the results of Agbor *et al.*, who reported an increased number of frequent adverse events in tobacco users and nonusers in Cameroon, including edentulousness (7·6 % and 0·9 %; *P* = 0·016), gingival recession (61·3 % and 46 43·0 %; *P* = 0·006) and tooth loss (38·7 % and 22·4 %; *P* = 0·008)^(68)^. Further investigations could explain the relationships between smokeless tobacco use and both undernourishment and dental problems in these women.

### Limitations

We used national data from the WHO STEPS survey, which aimed to study the prevalence and knowledge of common risk factors for noncommunicable diseases in the Burkinabè population aged 25–64 years and included nonspecific data on oro-dental health. The study design was cross-sectional in nature and could not establish causal relationships between variables. The use of self-reported dental problems rather than a validated tool (such as the Oral Health Impact Profile, which enables easier operationalisation of variables) to measure problems cannot provide specific parameters. Furthermore, the use of only chewing problems, pain or difficulty talking to assess dental problems is not an accurate representation of dental problems. A method based on clinical oral examination that objectively measures the dental conditions of respondents, thus measuring normative dental needs, would be useful in future studies. There was no analysis of food regimens that could interfere with nutritional status. The use of only BMI/weight but not overall nutritional parameters did not accurately reflect nutritional status. Data on the socio-economic level of households would also have allowed us to better understand the distribution of underweight. While these first nationally representative data from 2013 may no longer reflect the current situation, they provide a baseline that can be compared with future WHO STEPS survey data.

## Conclusion

The prevalence of underweight in rural Burkinabe women is among the highest in SSA and has geographical specificity due to the country’s urbanisation features and the national rainfall characteristics. Dental problems frequently increase underweight status. The respondents who most often experienced dental problems were women aged over 49 years, smokeless tobacco users and those with low BMI; these populations should be primary targets for public health prevention measures. Our study is the first to analyse the data of Burkinabè women and suggests that the association may be bidirectional as difficulty chewing or pain may lead to inadequate intake and weight loss, while inadequate intake and weight loss can lead to nutrient deficiencies manifesting in the oral cavity and weakening it, causing pain. Additionally, other factors, such as finances, diet or chronic diseases, may affect both nutrition/weight status and oral health. Nutrition interventions among rural women should take into account the dental condition of aged women to provide adequate food items. Further investigations using an appropriate design should highlight the specific relative risks for dental problems as well as underweight individuals.
